# Dispersed Oil Disrupts Microbial Pathways in Pelagic Food Webs

**DOI:** 10.1371/journal.pone.0042548

**Published:** 2012-07-31

**Authors:** Alice C. Ortmann, Jennifer Anders, Naomi Shelton, Limin Gong, Anthony G. Moss, Robert H. Condon

**Affiliations:** 1 Department of Marine Sciences, University of South Alabama, Mobile, Alabama, United States of America; 2 Dauphin Island Sea Lab, Dauphin Island, Alabama, United States of America; 3 Department of Biological Sciences, Auburn University, Auburn, Alabama, United States of America; University of Delaware, United States of America

## Abstract

Most of the studies of microbial processes in response to the Deepwater Horizon oil spill focused on the deep water plume, and not on the surface communities. The effects of the crude oil and the application of dispersants on the coastal microbial food web in the northern Gulf of Mexico have not been well characterized even though these regions support much of the fisheries production in the Gulf. A mesocosm experiment was carried out to determine how the microbial community off the coast of Alabama may have responded to the influx of surface oil and dispersants. While the addition of glucose or oil alone resulted in an increase in the biomass of ciliates, suggesting transfer of carbon to higher trophic levels was likely; a different effect was seen in the presence of dispersant. The addition of dispersant or dispersed oil resulted in an increase in the biomass of heterotrophic prokaryotes, but a significant inhibition of ciliates, suggesting a reduction in grazing and decrease in transfer of carbon to higher trophic levels. Similar patterns were observed in two separate experiments with different starting nutrient regimes and microbial communities suggesting that the addition of dispersant and dispersed oil to the northern Gulf of Mexico waters in 2010 may have reduced the flow of carbon to higher trophic levels, leading to a decrease in the production of zooplankton and fish on the Alabama shelf.

## Introduction

The explosion and sinking of the Deepwater Horizon drilling platform resulted in the release of an estimated 4.9 million barrels of crude oil and the application of >1.8 million gallons of dispersant into the waters of the northern Gulf of Mexico (nGOM) [1], [2], [3]. Most of the dispersant (1.07 million gallons) was applied to the surface with the rest applied at depth near the wellhead. This event stimulated intensive research in the region of the spill in an attempt to characterize the ecological response and impacts to both the oil and dispersant, especially in the deep water plume formed near the well head [4], [5], [6], [7].

Although much of the oil remained at depth and was prevented from reaching shore, significant oiling occurred from Louisiana to the western portion of the Florida panhandle [1], [2], [3], [8]. This surface slick included both oil and dispersants, mainly Corexit EC9500A [3], [9], and covered a large area of the highly productive shelf waters of the nGOM. It is these highly productive waters that support many of the fisheries that are economically important in the nGOM region [10]. In particular, the primary production by phytoplankton and consumption of terrestrially derived organic matter by heterotrophic prokaryotes provide the food and energy at the bottom of the pelagic food web to support the higher trophic levels.

Several studies have been carried out investigating the effects of oil contamination, sometimes along with chemical dispersants, on different members of the microbial communities. Often these studies are carried out on isolates under controlled conditions. The results of these types of studies highlight the individual response of different species to oil [11], [12], [13], different dispersants [14] and the combinations of oil and dispersants [15], [16], [17]. The responses to these additions are often not additive, with interactions between dispersants and oil resulting in larger or smaller responses than predicted from individual tests [15], [16]. The responses are also often dose dependent, with larger doses of oil and/or dispersant usually tending towards more toxic effects [13], [18]. Overall, the conclusions from previous studies suggest that some heterotrophic prokaryotes benefit and grow well with oil and/or dispersants, while others decrease in activity and abundance [19], [20], [21]. Small phytoplankton cells, such as *Prochlorococcus* and *Synechococcus*, were found to be more sensitive to contaminants than larger diatoms, but cultured isolates appeared to have higher tolerances compared to natural communities [13]. Studies of protists suggest that these organisms response positively to oil contamination, likely in response to increased prey [22], but the effects of dispersants are not well characterized.

One issue with using isolates or measuring individual group responses to oil and/or dispersants is that in the environment, the different groups are linked through complex direct and indirect interactions. A few studies have taken a wider approach and used large volume incubations to characterize the response of several different functional groups to oil, dispersant and/or dispersed oil to characterize how the whole food web responds to contamination. These studies have used static mesocosms [23], [24], [25] and flow through systems [26] to characterize phytoplankton, zooplankton, protist, prokaryote and, in one case, benthic [27] responses to contamination. Remarkably, although these studies were carried out from the Baltic to subtropical environments in Asia, similar patterns emerge. Phytoplankton appear to be strongly inhibited by dispersed oil, with the addition of dispersant alone having little effect compared to control samples [23]. Oil alone tends to decrease phytoplankton abundances or cause shifts in the community, but generally only at higher concentrations and in the absence of nutrient limitation [18], [24], [25], [27]. In all instances, prokaryote abundance increased in response to additions of oil, dispersant or dispersed oil, suggesting that all three treatments provide increased organic carbon that can be utilized by the prokaryote community. This often translated into higher number of heterotrophic flagellates [23], [24], suggesting this group responded to increased prey abundance. All treatments also appeared to have a consistent detrimental effect on mesozooplankton, with the largest effects on copepods [18], [24], [27]. As this group represents the link between the microbial food web and higher trophic levels, the loss of mesozooplankton would have a profound impact on the potential for fish production.

The previous studies were carried out in several different locations; however, the temperatures in most of these studies were <20°C. The surface slick in the Gulf of Mexico during the summer of 2010 would have experienced significantly higher temperatures, 25–30°C, which may alter the response of the microbial community to the influx of oil and dispersants during the Deepwater Horizon spill. We used a mesocosm approach to characterize the response of the microbial food web in the waters of the Alabama shelf to the input of oil and application of dispersants during the Deepwater Horizon oil spill. Using large volume incubations, we were able to ask how oil, dispersant and dispersed oil may have influenced the cycling of carbon between viruses, prokaryotes, phytoplankton and microzooplankton.

## Methods

Mesocosm incubations were carried out in early (June) and late (August) summer 2011 at the mesocosm facility at the Dauphin Island Sea Lab. Five 200 l barrels lined with Teflon bags were each placed in five 2000 l tanks (25 barrels total). Each barrel had large holes cut in the side to allow gentle mixing by flowing water in the large tank. In the middle of each barrel a pipe extended to mid-water depth, with an air bubbler at the bottom to prevent stratification and hypoxia. Samples were collected using a peristaltic pump and Teflon tubing inserted into the center tube, thereby avoiding disturbance of the surface layer of the mesocosms. The mesocosms were protected from direct sunlight by a roof, although the sides of the facility were open. Some lighting around the structure prevented complete darkness, but the use of bright overhead lights directly over the mesocosms was restricted.

Water was pumped into the mesocosms through an intake tube extending south of Dauphin Island. The mesocosms were filled at high tide and water was continually flowing in the large tanks to help maintain *in situ* temperatures. In June, the mesocosms were filled ∼6 h before the start of the experiment, while in August, they were filled ∼24 h before the start. The intake for the system has a 6.35 mm screen to exclude large organisms.

Five different treatments were applied with 5 replicates of each treatment, one per large tank. These included a no addition control (Control), the addition of 3.0 mM of carbon as glucose as a carbon addition control (Glucose), the addition of dispersant (Corexit 9500A) representing ∼0.7 mM of carbon (Dispersant), the addition of MC 252 oil (Oil) representing 30 mM of carbon and the addition of both oil and dispersant together at the same concentrations as above (30.7 mM carbon, Dispersed Oil). The dispersant was added in a 1∶20 v/v ratio to the oil. All the additions were completed in 35 to 45 m with t = 0 set as the time the last compounds were added. Approximately 2 h before the addition of the compounds, samples were collected from 5 of the 25 mesocosms. Subsamples for biomass were collected over 5 d, with more intense sampling occurring earlier in the experiment. Before additions and at 1 and 3 d after additions short incubations were run to measure cell growth rates and virus production. In August, one of the Teflon bags in the Dispersed Oil treatment developed a leak, resulting in mixing with the surrounding water. This mesocosm was not included in any of the analyses, resulting in n = 24 for this experiment.

### Physical Parameters

The temperature (°C), salinity (ppt) and dissolved oxygen (DO, mg l^−1^) in each mesocosm were measured daily using a YSI Pro2030 handheld probe. The probe was cleaned following exposure to oil and dispersed oil according to the protocols from YSI. The data from t = 0 in June was accidently deleted before being recorded, so data presented below represents t = 1 d. Water characteristics were measured at approximately the same time every day.

### Nutrient Concentrations

The concentration of NO_2_^−^, NO_3_^−^, NH_4_^+^, TDN and PO_4_^−3^ were determined for 5 mesocosms at t = 0, and all mesocosms at all subsequent time points. Samples were pumped from the central tube directly into an acid-washed collection bottle. The water was then filtered through pre-combusted (500°C for 4 h) 0.7 µm Whatman GF/F filters, and dissolved nutrients determined in the filtrate. Total dissolved N (TDN) was analyzed by persulfate oxidation [28], [29], NO_3_^−^ by the spongy cadmium (Cd) method, and NO_2_^−^ and PO_4_^3−^ were measured on a Skalar SAN+ nutrient autoanalyzer [30]. NH_4_^+^ was determined using sodium hypochlorite and fluorometric detection [30]. During analysis, the conversion of NO_3_^−^ to NO_2_^−^ by Cd catalyst was monitored and columns regenerated if reduction efficiency was <97%.

### Characterization of Oil and Dispersant Droplets

To determine the relative abundance of oil and dispersant droplets, samples were collected for analysis by a FlowCAM (Fluid Imaging Technologies, Yarmouth, ME). Samples (1 ml) from each mesocosm were prescreened through a 125 µm mesh and imaged in a 100 µm deep flow cell using a 10× Olympus UPlan FLN infinity objective lens. The flow rate was 0.5 ml s^−1^ with data collected using the auto-triggering setting. Raw data was filtered to count particles between 3 and 20 µm with a circle fit of 0.9 to 1 and a compactness window of 1.0 to 1.5. The filter (combining size and shape) was determined empirically from examination of multiple oil and dispersant data sets and then applied to the images collected for all treatments.

### Biomass Measurements

At each time point samples were collected to determine the abundance of viruses, prokaryotes, diatoms, dinoflagellates, ciliates and heterotrophic nanoflagellates (HNFs). Samples for viruses and prokaryotes were fixed in 0.5% final concentration of EM grade glutaraldehyde and flash frozen in liquid nitrogen within 30 m. Samples were stored at −80°C until analysis with flow cytometry [31], [32]. Briefly, samples were thawed in small groups at 37°C then diluted with filtered TE (100× for viruses and 10× for prokaryotes) [33], [34]. Virus samples were stained with SYBR Green (1∶20 000 final dilution) for 10 m at 80°C and then run on a FACSCalibur flow cytometer (BD Biosciences, San Jose, CA). Prokaryotes were stained for 15 m at room temperature and data collected for 3 m. Yellow-green 1 µm beads (Polysciences, Inc.) were added to each sample as an internal quality control. Counts were obtained over a specific time (2 m) and converted to abundances using the empirically determined flow rate for the medium setting. FSC files were imported into GateLogic (Inivai Technologies, Mentone, Australia) and analyzed to determine the abundance of viruses or prokaryotes using optimized gates. Small adjustments of the gates were made by eye as the prokaryote populations occasionally shifted. The abundance of cells or viruses in each gate was converted into l^−1^ estimates using the measured flow rates and the time of data collection. Using average estimates of 30 fg C cell^−1^ and 0.2 fg C virus^−1^, abundances were converted into µg C l^−1^ [35].

Samples were collected for analysis of phytoplankton and ciliates in a glass jar. These samples were preserved with acidic Lugol's and counted using an inverted microscope [36]. For each sample, 11 ml was settled in a chamber slide for 45 min before being counted under a 40× objective with an ocular grid. Dinoflagellates, diatoms and ciliates were counted separately, although not identified further, and counts converted to cells l^−1^. In June, HNFs were fixed in 0.5% EM grade glutaraldehyde for 30 m and then filtered onto a black 0.8 µm polycarbonate filter, stained with DAPI (0.005 mg ml^−1^), mounted on a slide and stored at −20°C until counting on an Olympus B-2 microscope under UV light using a 40× objective. In August, the flagellates were preserved in alkaline Lugol's with buffered formaldehyde and a higher concentration of DAPI was used (1.0 mg ml^−1^) [37]. All flagellate counts were converted to cells l^−1^. For the dinoflagellates, diatoms, ciliates and HNFs, several cells were measured to obtain an average volume based on standard geometric shapes. No corrections were made for shrinkage of cells, therefore volumes are minimum estimates. Volumes were used to calculate an average carbon/cell estimate to change cell numbers into µg C l^−1^ [38].

At the beginning and end of each experiment, samples were filtered to obtain mesozooplankton abundances. Because of the limited volumes, all replicates were pooled at the end. Analyses of the samples from the beginning of the experiments suggest that the abundance of zooplankton was close to or below detection limits, and were unlikely to play a major role in these incubations.

### Prokaryote Growth and Virus Production

To determine the growth rates of prokaryotes and rates of virus production simple dilution incubations were carried out in parallel to the large mesocosms. These experiments were performed at t = 0, 1 and 3 d. Prior to filling the mesocosms, 20 l of water was collected from the intake pipe and filtered through a 142 mm 0.7 µm glass fiber filter followed by a 142 mm 0.2 µm Durapore filter (Millipore) to remove cells. The water was then passed through a 30 kDa tangential flow filter (Pall Corporation, Port Washington, NY) and the cell and virus free filtrate was collected. For the incubations, 5 ml of sample was collected from each mesocosm and added to 45 ml of the filtrate in polyethylene bags with wire closures (Fisherbrand). Sub samples were collected at 0, 3 and 6 h and processed as described above for prokaryote and viruses abundances. Growth and virus production rates were calculated from the regression of ln(cell number or virus number) vs time over 6 h. Regressions with p-values<0.1 were considered significant.

### Data Analysis

The initial characteristics of the mesocosms were compared between June and August using nonparametric Welch tests. Values for t = 0 were used for nutrient concentrations and biomass abundances. For temperature, salinity and DO, comparisons were carried out using data from the Control mesocosms collected at t = 1 d.

The effects of treatment on physical characteristics, nutrients and the abundance of different groups of microbes were determined using the multivariate repeated measures analyses based on the MANOVA function in JMP 9.0 (SAS Institute, Inc.). Significant between-subject effects represented different effects of the treatment which was further investigated using the Canonical Centroid Plot to identify which treatments were significantly different from each other. Within-subject analyses were investigated to determine if the interaction between time and treatment was significant. Significant tests, based on the Pillai's Trace value, were further investigated using the Canonical Centroid Plot to determine if the treatments had different effects over time.

The effects of the treatments over time on the contributions of the six microbial groups to the total community were analyzed using analysis of similarity (ANOSIM) in Primer 6 (Primer-E). Biomass estimates (µg C l^−1^) were fourth root transformed and a Bray-Curtis similarity matrix generated for each mesocosm at t = 0, 1 and 3 d. A two way-crossed analysis using Treatment and Time was run for each experiment separately. If Global R values for either Treatment or Time were determined to be significant (R>0.3 and p<0.05), then pairwise comparisons were carried out to determine which times or treatments were different. Pairwise tests were significant if R>0.3 and p<0.017 (Time) or <0.006 (Treatment). Large values of R indicate significantly different communities.

## Results

The starting conditions for the two experiments differed in terms of the community composition (biomass of different groups) and nutrient status (Table S1). The June community had a larger biomass of primary producers, both diatoms and dinoflagellates, compared to the large contribution of heterotrophic groups to the biomass in August (Figure S1). Based on nutrient concentrations and the N∶P ratio, the June community was PO_4_^−3^ limited at the start of the experiment, while the August community did not appear to be nutrient limited.

**Figure 1:**
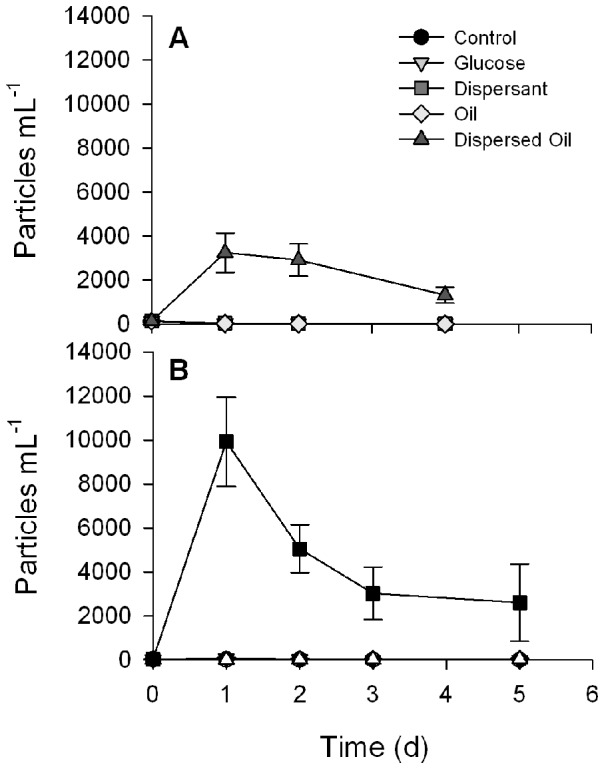
Number of spherical particles 3–20 µm in diameter in each treatment over time. June (A) and August (B) both show a rapid increase in particles in the Dispersed Oil treatment at t = 1 d, with a decrease overtime. This decrease was likely due to small particles clumping together and becoming larger than 20 µm. Small differences were observed for the other treatments, but they were not significantly different from each other.

Most of the oil added to the mesocosm remained on the surface as a slick. The low mixing of the oil into the water column resulted in dissolved organic carbon (DOC) concentrations in the Oil treatments that were similar to the Control treatments over time. The highest DOC was measured in the Glucose treatments while the Dispersant and Dispersed Oil were similar, but lower than the Glucose addition (Figure S2). The dispersed oil was completely mixed in the mesocosm after 6 h, with only a small amount of slick visible on the surface. The water samples from this treatment had a distinctive yellowish color and small droplets were visible on HNF and protist slides. FlowCAM analysis clearly shows significant difference in the number of small, spherical particles present in the Dispersed Oil treatment compared to all other treatments (Figure 1), indicating good dispersion of the oil.

**Figure 2:**
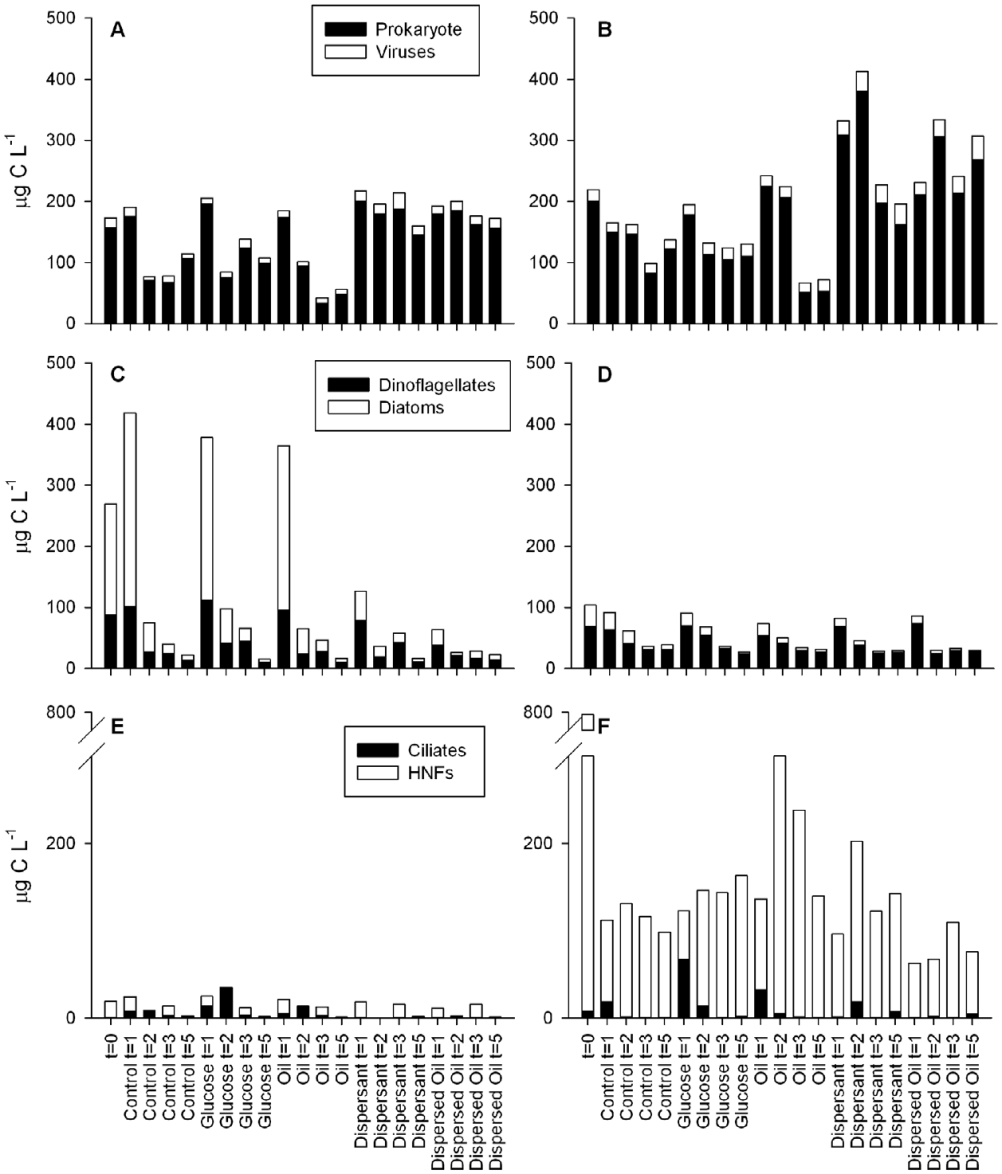
Distribution of carbon in the six microbial groups over time for each of the five treatments. Values shown are averages for the five replicates. Values from the June experiment are shown on the left for Prokaryotes and Viruses (A), Dinoflagellates and Diatoms (C) and Ciliates and HNFs (E). August values on the right (B, D and F). In June (E), HNF values were not obtained at t-2 and 5 d, but all data was collected for August (F).

The biomass of the six different microbial groups changed over time, with significantly different patterns due to treatment effects (Table S2). The distribution of carbon between these groups changed over time as the community structure changed (Figure 2). Generally, the patterns observed in the Glucose and Oil treatments were more similar to the Control treatment, while the patterns in the Dispersant and Dispersed Oil treatments were more similar to each other (Figure 2). The main observation in both June and August was a decrease in the biomass of prokaryotes in the Control, Glucose and Oil treatments with an increase in the Dispersant and Dispersed Oil (Figure S3). Dinoflagellates and diatoms decreased in all treatments in both experiments over the course of 5 d; however the decreases were more rapid in the Dispersant and Dispersed Oil treatment and transient increases were detected in Control, Glucose and Oil treatments in June (Figure S4). Ciliates, which were low at the start of the experiments, significantly increased in the Glucose treatments, with smaller, but significant increases in the Oil and Control treatments at the same time. The peak in June was detected at 2 d compared to 1 d in August. In June, the biomass of HNFs was low compared to August and remained low throughout the experiment with no significant effects of treatment (Figure S5). The August experiment started with a very high biomass of HNFs which decreased rapidly by 1 d, but this group represented a large proportion of the microbial biomass throughout the experiment. Significantly more HNFs were detected in the Oil treatments compared to the Dispersed Oil treatments, although neither treatment was significantly different from the Controls.

ANOSIM analysis using the six different groups and three time points indicated significant effects of both time and treatment on community structure (Table 1). The community structure in all treatments changed rapidly, with little similarity between the communities at 0 and 3 d. Changes in the community structure were also affected by the treatment. Over time, the community structure in the Dispersant and Dispersed Oil treatments were not significantly different from each other, but both communities differed significantly from the communities in the Control, Glucose and Oil treatments. In August, the Control, Glucose and Oil treatments did not result in significantly different community structures, but significant differences were detected between the Glucose and Oil treatments in June (Table 1).

**Table 1:** ANOSIM results from a two-way crossed analysis testing the effects of treatment and time on the community structure.

	June		August	
	R	p-value	R	p-value
Time, Global R	**0.81**	0.001	**0.92**	0.001
0 d vs 1 d	**0.84**	0.008	**0.99**	0.008
0 d vs 3 d	**1.00**	0.008	**1.00**	0.008
1 d vs 3 d	**0.80**	0.001	**0.90**	0.001
Treatment, Global R	**0.58**	0.001	**0.45**	0.001
Control vs Glucose	0.27	0.018	0.13	0.101
Control vs Oil	0.15	0.072	0.16	0.067
Control vs Dispersant	**0.98**	0.001	**0.67**	0.001
Control vs Dispersed Oil	**0.75**	0.001	**0.66**	0.002
Glucose vs Oil	**0.53**	0.001	0.21	0.015
Glucose vs Dispersant	**0.76**	0.001	**0.67**	0.001
Glucose vs Dispersed Oil	**0.73**	0.001	**0.74**	0.001
Oil vs Dispersant	**0.99**	0.001	**0.67**	0.001
Oil vs Dispersed Oil	**0.840**	0.001	**0.70**	0.001
Dispersant vs Dispersed Oil	0.334	0.001	−0.03	0.505

Global R values represent the overall significance of the test for either time or treatment while pairwise R values indicate which factors differed. Large R values indicate more differences between communities, while small R values indicate more similarity. P-values are indicated for each test.

**Figure 3:**
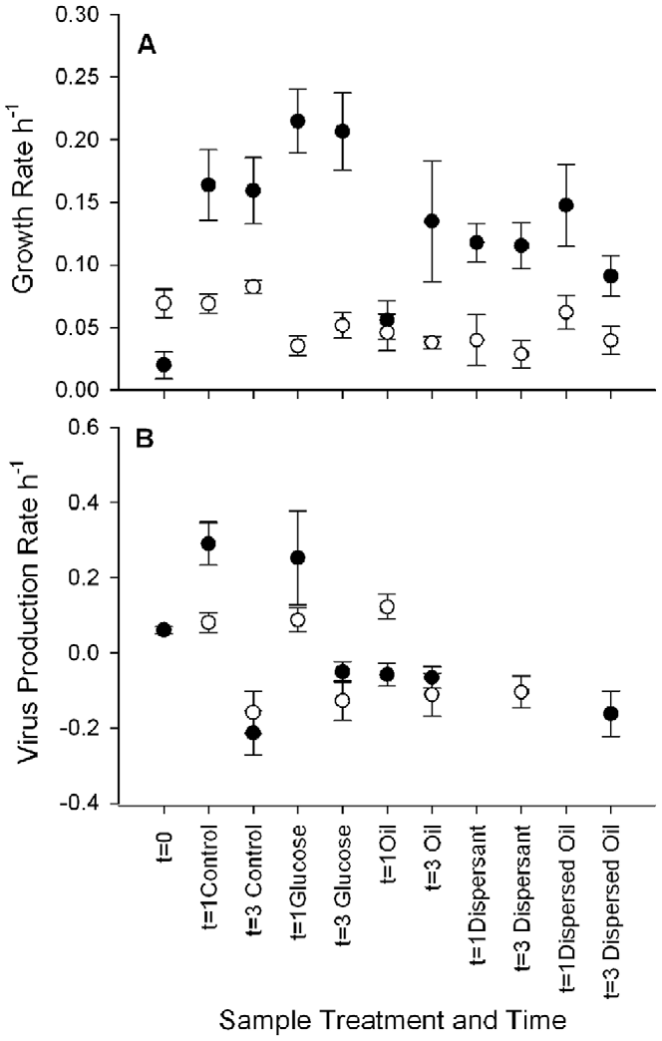
Prokaryote growth rates (A) and virus production rates (B) from incubations at t = 0, 1 and 3 d. Values presented are the slope (h^−1^) from the regression of ln(abundance) vs time (p<0.1), with the standard error of the estimate. Solid circles are from June and open circles are from August. Missing symbols in the virus production plot indicate non-significant regressions.

At three time points (t = 0, 1 and 3 d) incubations were carried out to measure the prokaryote growth rate and virus production rate. Growth in June at t = 0 was significantly lower than in August, but growth rates at t = 1 and 3 d was higher in all treatments in June compared to t = 0 (Figure 3A). The highest growth rates in June were detected in the Glucose treatment, however growth was stimulated in all treatments compared to t = 0. In August, only the Control treatments were equal to the t = 0 rate, with all other treatments significantly lower at 1 and 3 d. In June, low rates of virus production were detected at t = 0, with significantly higher rates detected in the Control and Glucose treatments at 1 d (Figure 3B). A similar pattern was seen in August; although no virus production was detected at t = 0, low rates were detected at 1 d in the Control, Glucose and Oil treatments. No virus production was detected in the Dispersant or Dispersed Oil treatments, with virus loss commonly detected at 3 d in all treatments.

**Figure 4:**
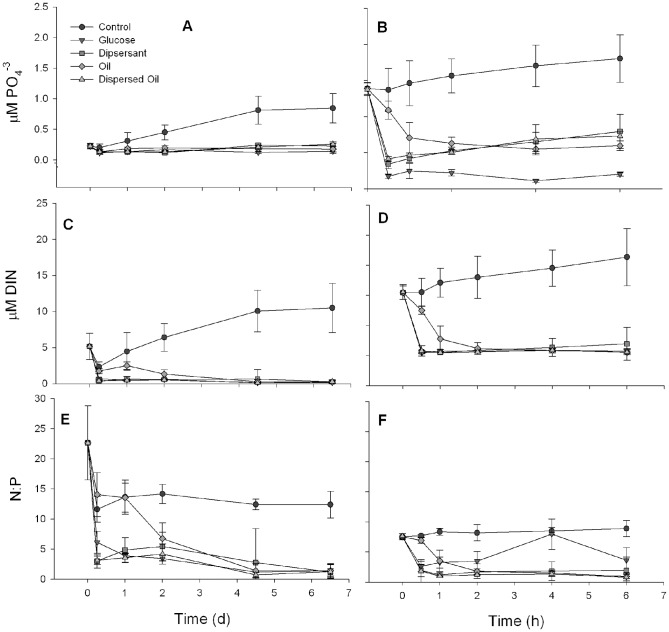
Concentration of PO_4_^−3^ (A, B), total DIN (NO_2_^−^+NO_3_^−^+NH_4_^+^, C, D) and N∶P (E, F). June (A, C and E) and August (B, D and F) showed similar patterns even though the starting conditions were different. Error bars are standard deviations of the means.

Although the concentration of DIN and PO_4_^−3^ were significantly different at the start of the June experiment compared to August (Table S1), similar patterns in nutrient usage were detected in both experiments (Figure 4). There was a significant effect of the addition of all substrates on the utilization of both DIN and PO_4_^−3^ compared to the Control (Table S3). In the Control treatments, DIN and PO_4_^−3^ increased over time, while they decreased in all of the other treatments. There were some differences in the rates of drawdown of nutrients, with significantly slower decreases in the Oil treatments. The N∶P ratio decreased in all treatments, with changes in the Control treatments due to differences in regeneration rates of DIN and PO_4_^−3^. In the other treatments, higher utilization of DIN compared to PO_4_^−3^ resulted in lower N∶P ratios.

## Discussion

The similar patterns of biomass change and nutrient utilization in the two experiments suggests that there are distinct differences in how these additives are perceived by the planktonic microbial community. The dispersant-associated carbon that enters the food web is not equivalent to carbon in the form of glucose or even oil alone. The changes in the community structure, either because of exploitation or toxicity effects, likely have significant effects on higher trophic levels including fisheries production.

Compared to the Control treatment in which no additions were made, few changes in the community structure were observed when glucose or oil was added. The lack of strong ecosystem effects of oil may be due to the decreased bioavailability of the oil throughout the water column compared to studies that used the water accessible fraction (WAF) [24] or higher concentrations of oil (>1000 ppm) [18]. Most of the oil added to our mesocosms remained as a slick and slowly mixed into the water column. Thus, although we added the oil at 500 ppm, oil concentrations were likely higher in the upper portion of the mesocosms. Large, short-term effects have been correlated with higher oil concentrations that was present in our experiments (>1000 ppm, [18]), therefore exposure to lower concentrations may have reduced potential toxic effects or delayed any impacts beyond the timeframe of the experiments. In contrast to what was experienced during the Deepwater Horizon incident where multiple pulses of oil moved over the Alabama shelf [39], the communities in our experiment were exposed to a single dose of oil over a short time. Multiple exposures to oil alone, or with dispersants, would likely result in the microbial community responding more strongly than measured in the experiments described here.

Although patterns in biomass changes were consistent over the course of the experiment in the Control, Glucose and Oil treatments (Figure 3), differences in the nutrient concentrations suggest that different biochemical and metabolic processes were occurring (Figure 4). The increase in DIN and PO_4_^−3^ in the control treatments suggest that the microbial community was remineralizing organic matter and releasing inorganic nutrients. The mortality of dinoflagellates, diatoms and HNFs may have provided the organic matter necessary for the prokaryotes to grow. In treatments where glucose or oil was added, a strong decrease in the concentrations of inorganic nutrients suggests the communities were utilizing the C-rich substrates. The higher growth rates of prokaryotes detected when glucose was added suggest that glucose was a more easily utilized compared to the oil. The lag in the drawdown of nutrients in the Oil treatments, along with a lag in an increase in growth rate in June, may indicate that organisms capable of utilizing the oil were at low abundances, and did not begin to efficiently use the substrate until 2–3 days into the experiment.

In the oil and glucose treatments, the additional carbon added appears to be transferred to the ciliates, based on a decrease in the prokaryote biomass and an increase in the ciliate biomass, even as the growth rates suggest actively growing prokaryote communities. Based on the estimates of cell growth, the biomass of prokaryotes in the glucose treatment should have been higher than any other treatment, so coupling between growth and grazing must have been strong. Estimated growth rates of prokaryotes in the oil treatments were lower than in the control and glucose treatments, but the larger decrease in prokaryote biomass along with an increase in ciliate biomass suggests grazing by microzooplankton was still an important process. In these experiments, it appears that grazing exceeded growth and was a more important process than viral lysis in controlling the abundance of prokaryote cells.

Dispersant treatments, with and without oil, had significant effects on the biomass of most of the microbial groups compared to both the control and the addition of glucose and oil alone. The most immediate impact was observed with the eukaryotes, with significant decreases in dinoflagellates during June and in diatoms and ciliates in both experiments. The magnitudes of the impacts were not identical; suggesting that some of the responses observed with dispersed oil was due to the interaction between the oil and dispersant on food web dynamics. The largest positive impact was on the biomass of the prokaryotes. The decrease in nutrients and positive growth rates suggest that the prokaryotes were able to use the dispersant and dispersed oil as a carbon source and that increased biomass could be due to reduced grazing by ciliates and dinoflagellates. Virus production was not detected in either dispersant treatment at any time, suggesting viral lysis was not a factor in these incubations. The slight increased carbon in viruses in dispersant treatments suggests that mechanisms responsible for the removal of viruses were disrupted by the addition of dispersant and dispersed oil. One possibility is that protists are grazing on viruses in this system and the decrease in ciliates, dinoflagellates and HNFs may have reduced removal of viruses [40]. Alternatively, viruses may be removed by binding to other particles (i.e. sediment, cells) and the addition of dispersants may alter the interactions between these particles and viruses.

The increase in prokaryote biomass and the negative impact of dispersed oil on the phytoplankton communities agrees with previous studies, but the strong response to dispersant alone is novel. In agreement with previous studies, we detected an impact on phytoplankton and non-flagellate protists in our incubations with dispersed oil [23], [25], [27]. A consistent response of the microbial community to dispersed oil appears to be an uncoupling in the transfer of energy through grazing and the reduction of primary production. In the previous studies, increases in HNFs were detected, which may have been responsible for grazing some of the increased prokaryote biomass [23], [27]. In our study, the ciliates appeared to be the dominant grazers in the community, and the HNFs did not appear to respond positively or negatively to any of the treatments. The negative response of phytoplankton and ciliates in our experiments differs from a previous study that detected no effect of dispersant alone on these groups [23].

The difference in the response may be due to differences in the dispersants used, Corexit 9500 in our study compared to Corexit 9527 in the previous study [23] or the difference in temperature in the studies. In the previous study, large (∼63 000 l) floating mesocosms were used to characterize the effects of Corexit 9527 by itself and with Alaskan crude oil. In the dispersed oil treatment, decreases in phytoplankton and zooplankton were detected compared with a no addition control, with increases in prokaryotes and flagellates. In the dispersant only treatment, the only difference between the treatment and control was a small increase in the prokaryote activity and abundance. Different formulations of dispersants have been shown to have different effects on isolates in culture [14], so it is not unexpected that whole microbial communities may respond differently to different dispersants. It is also possible that the higher temperatures in our study (∼30°C) compared to Saanich Inlet, BC, Canada (∼12–15°C) may have resulted in different interactions between the dispersant and the microbial cells. In a study looking at the interaction between dispersed oil at low temperatures (∼−1.6°C), high settling of oil and poor dispersion was observed [26]. This suggests that temperature would affect how dispersants might interact with the oil, water column and the organisms in it.

**Figure 5:**
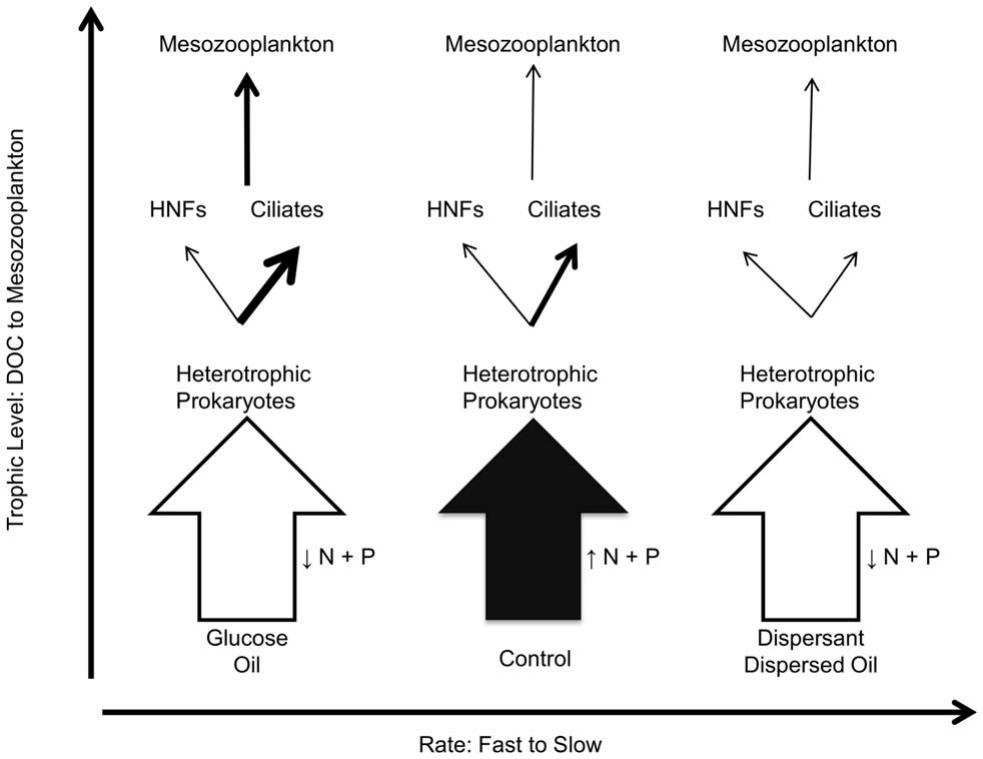
The effects of different DOC sources on the microbial food web. The addition of Glucose or Oil rapidly decreases N+P, increasing the biomass of heterotrophic prokaryotes, resulting in a large increase in ciliates and a predicted transfer of biomass to mesozooplankton. Addition of Dispersant or Dispersed Oil also results in an increase in heterotrophic prokaryotes and a drawdown of N+P, but this biomass does not result in a ciliate response and transfer to higher trophic levels is blocked. In the Control, organic matter is remineralized, releasing N+P, and some biomass from the heterotrophic prokaryotes transfers through ciliates to mesozooplankton.

The results of these mesocosm studies have implications for higher trophic levels dependent on the pelagic microbial food web. These incubations suggest that the application of dispersants results in a shift in the structure of the microbial community which could interrupt the functioning of the microbial food web (Figure 5). In turn, these alterations to trophic pathways could act as a shunt, diverting carbon from higher trophic levels. The addition of dispersant and dispersed oil resulted in a rapid decrease in the biomass of primary producers, especially for diatoms. A large negative impact on primary producer biomass would decrease the carbon available to larger microzooplankton and the mesozooplankton that graze directly on phytoplankton. While glucose and oil additions resulted in an increase in heterotrophic prokaryote growth rates this increase in biomass appeared to be transferred to higher trophic levels through classical food web mechanics. However, a bottleneck results in the transfer of energy due to dispersants, resulting in an increase in heterotrophic prokaryote biomass. The fate of this trapped carbon is likely release through respiration, decreasing the overall energy of the system. Understanding the responses of microbes to dispersant-mediated changes to the food web structure is essential in interpreting management responses to future spills and fisheries resources in coastal regions.

## Supporting Information

Figure S1 **Biomass of the six microbial groups at t = 0 in June and August.** Means with standard deviations are shown.
(TIF)

Figure S2 **Concentration of DOC in the mesocosms over time.** Mean DOC concentrations with standard deviations are shown for each treatment over time for June (A) and August (B).
(TIF)

Figure S3 **Biomass for prokaryotes (A/B) and viruses (C/D) over time, by treatment.** Means and standard deviations are shown for each treatment for June (A/C) and August (B/D).
(TIF)

Figure S4 **Biomass for dinoflagellates (A/B) and diatoms (C/D) over time, by treatment.** Means and standard deviations are shown for each treatment for June (A/C) and August (B/D).
(TIF)

Figure S5 **Biomass for ciliates (A/B) and HNFs (C/D) over time, by treatment.** Means and standard deviations are shown for each treatment for June (A/C) and August (B/D).
(TIF)

Table S1 **Means and standard deviations for the starting conditions for the two experiments along with the p-value from the non-parametric Welch test.**
(DOCX)

Table S2 **P-values from MANOVAs carried out comparing the biomass of each group using Treatment as the factor and repeated measures analysis with time.**
(DOCX)

Table S3 **P-values from MANOVAs carried out as above for nutrients.**
(DOCX)
