# Supplementary figures and images for: Dispersed Oil Disrupts Microbial Pathways in Pelagic Food Webs
Source: PLoS One. 2012 Jul 31;7(7):e42548. doi: 10.1371/journal.pone.0042548 (PMC3409195; doi:10.1371/journal.pone.0042548)

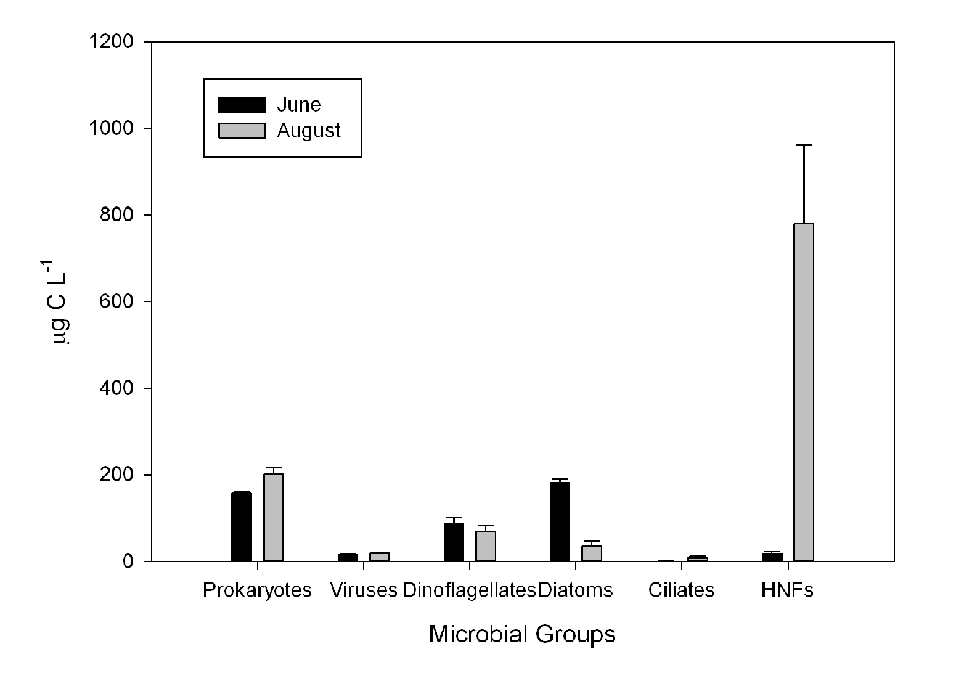

Supplement: Figure S1 — Biomass of the six microbial groups at t = 0 in June and August. Means with standard deviations are shown. (TIF) [file pone.0042548.s001.tif]

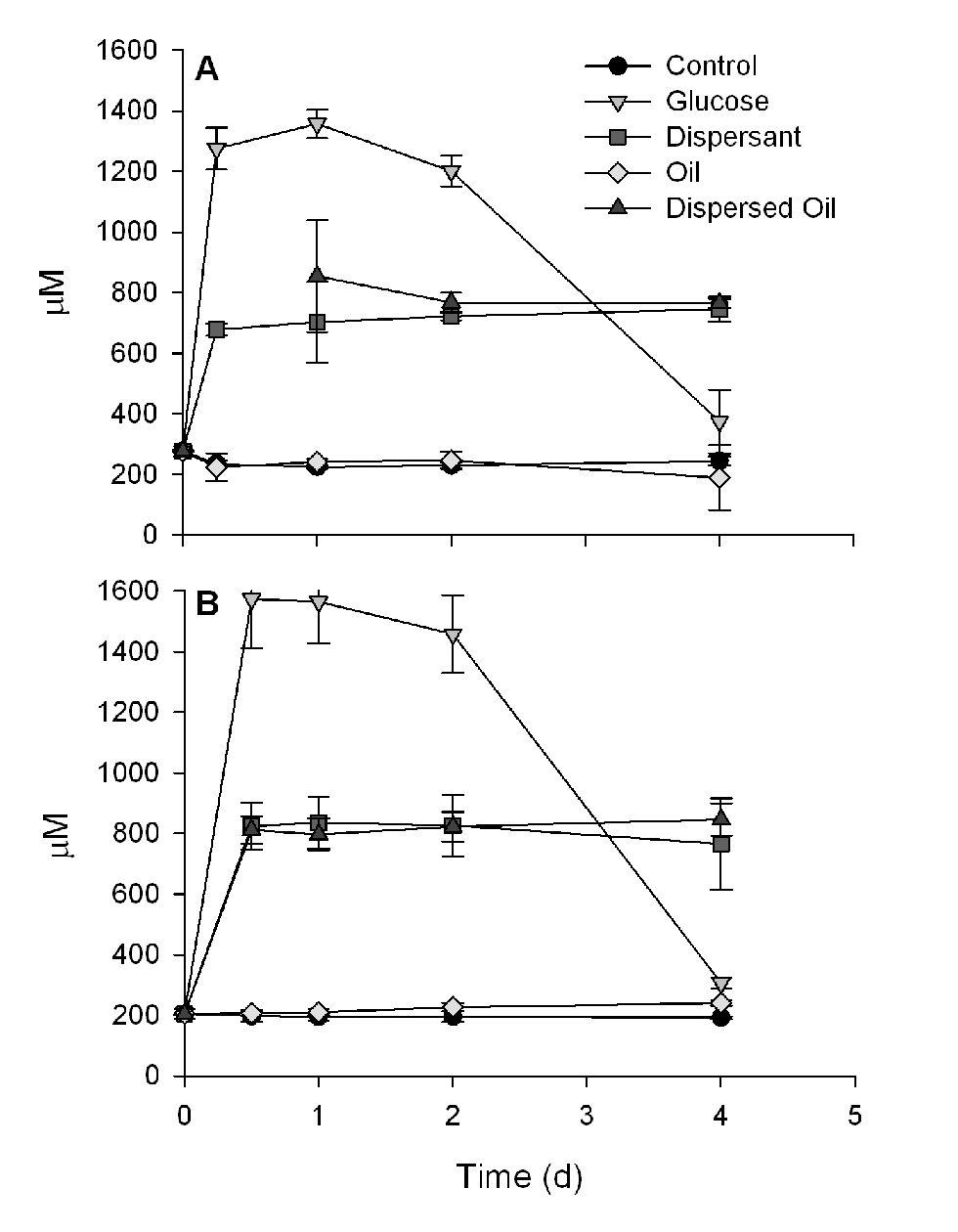

Supplement: Figure S2 — Concentration of DOC in the mesocosms over time. Mean DOC concentrations with standard deviations are shown for each treatment over time for June (A) and August (B). (TIF) [file pone.0042548.s002.tif]

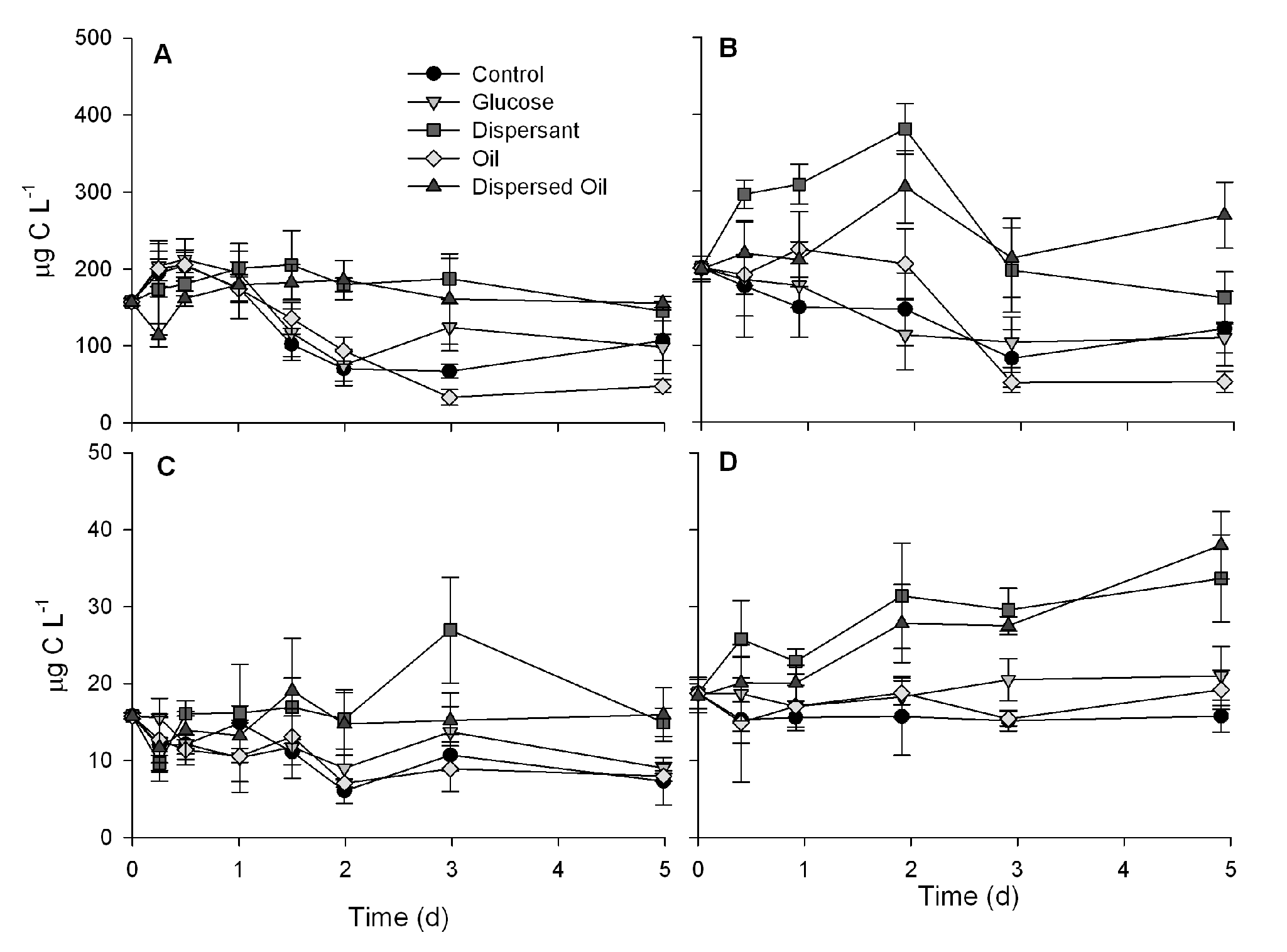

Supplement: Figure S3 — Biomass for prokaryotes (A/B) and viruses (C/D) over time, by treatment. Means and standard deviations are shown for each treatment for June (A/C) and August (B/D). (TIF) [file pone.0042548.s003.tif]

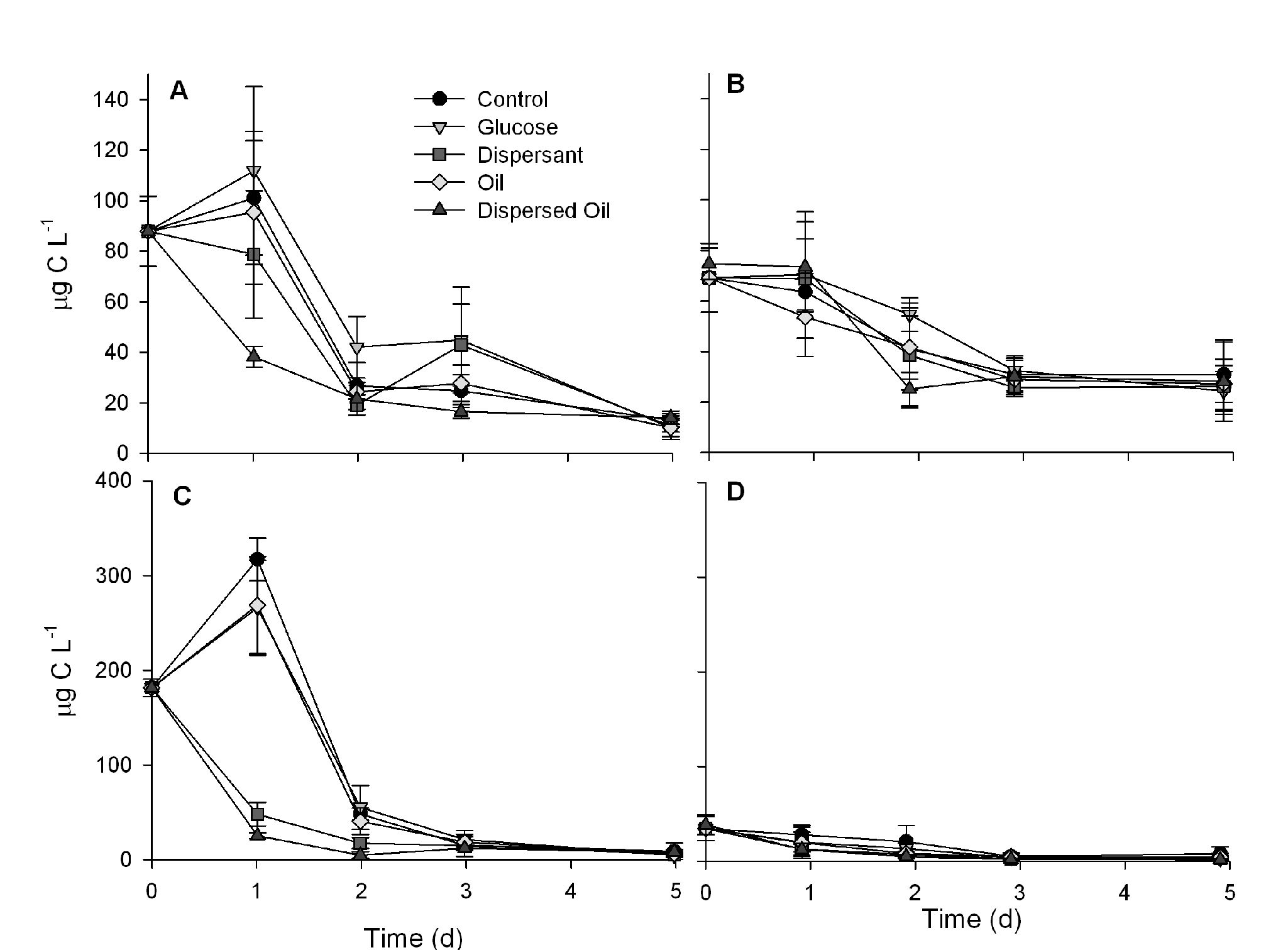

Supplement: Figure S4 — Biomass for dinoflagellates (A/B) and diatoms (C/D) over time, by treatment. Means and standard deviations are shown for each treatment for June (A/C) and August (B/D). (TIF) [file pone.0042548.s004.tif]

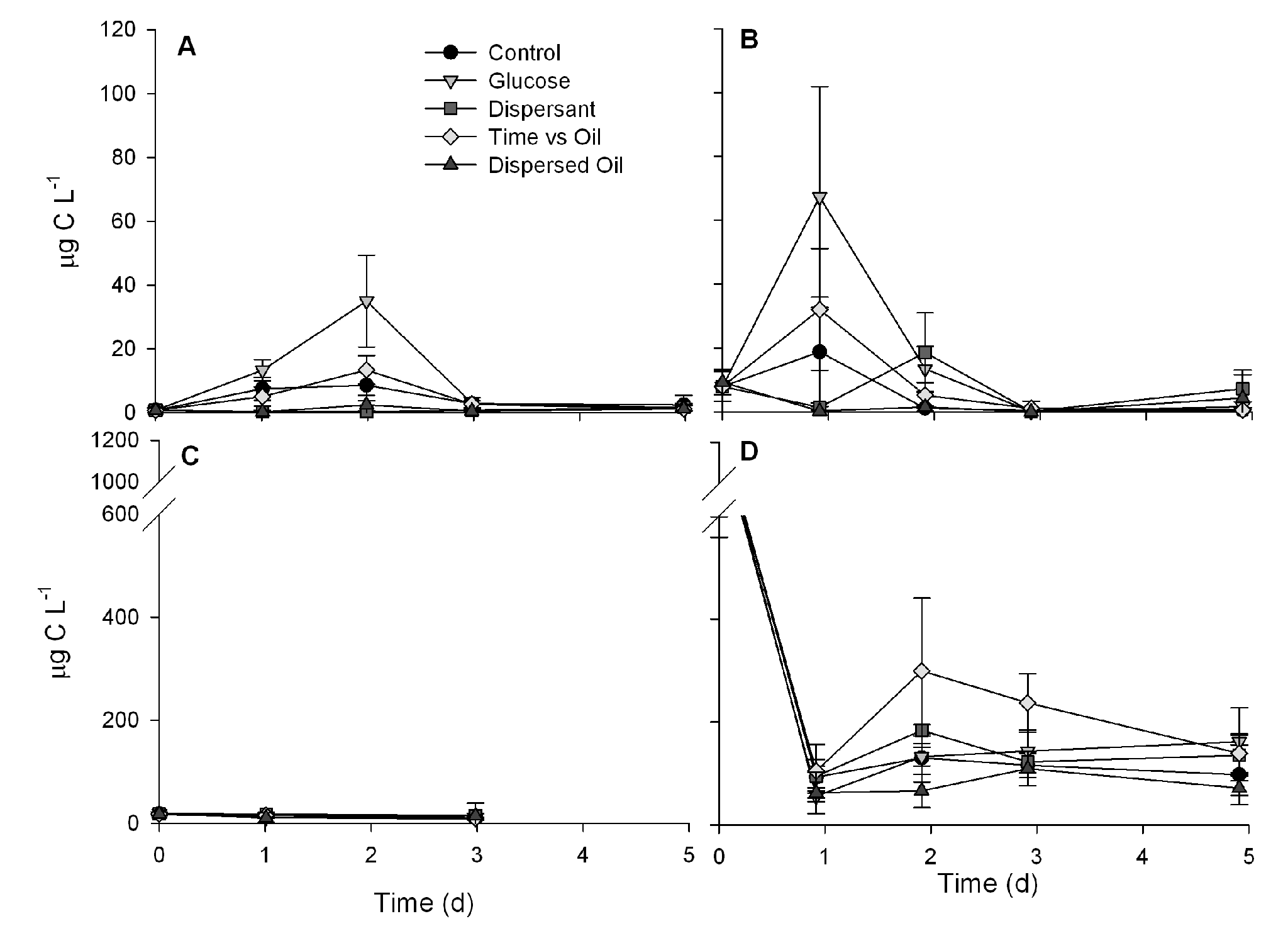

Supplement: Figure S5 — Biomass for ciliates (A/B) and HNFs (C/D) over time, by treatment. Means and standard deviations are shown for each treatment for June (A/C) and August (B/D). (TIF) [file pone.0042548.s005.tif]
