# Supplementary material for: Dispersed Oil Disrupts Microbial Pathways in Pelagic Food Webs
Source: PLoS One. 2012 Jul 31;7(7):e42548. doi: 10.1371/journal.pone.0042548 (PMC3409195; doi:10.1371/journal.pone.0042548)
Supplement: Table S1 — Means and standard deviations for the starting conditions for the two experiments along with the p-value from the non-parametric Welch test. (DOCX) [file pone.0042548.s006.docx]

**Table S1.** Means and standard deviations for the starting conditions for the two experiments along with the p-value from the non-parametric Welch test.

|  | June | August | p-value |
| --- | --- | --- | --- |
| Temperature (°C)^a^ | 30.26 (0.7) | 30.84 (0.11) | 0.1372 |
| Salinity (ppt)^a^ | 22.56 (0.65) | 21.94 (1.38) | 0.4005 |
| DO (mg l^-1^)^a^ | 4.85 (0.49) | 4.45 (0.38) | 0.1907 |
| Prokaryote Cells | 5.24×10^9^ (1.61×10^8^) | 6.68×10^9^ (4.96×10^8^) | **0.0018** |
| Viruses | 7.89×10^10^ (1.86×10^9^) | 9.37×10^10^ (1.02×10^10^) | **0.0302** |
| Dinoflagellates | 1.87×10^5^ (2.95×10^4^) | 1.47×10^5^ (2.89×10^4^) | **0.0314** |
| Diatoms | 4.74×10^5^ (2.43×10^4^) | 9.04×10^4^ (3.31×10^4^) | **0.0001** |
| Ciliates | 3.18×10^3^ (3.18×10^3^) | 3.31×10^4^ (1.91×10^4^) | **0.0237** |
| Flagellates | 5.10×10^6^ (1.02×10^6^) | 2.17×10^8^ (5.04×10^7^) | **0.0007** |
| NO_2_^-^ | 0.16 (0.03) | 0.65 (0.05) | **0.0001** |
| NO_3_^-^ | 2.77 (1.87) | 2.82 (0.30) | 0.9544 |
| NH_4_^+^ | 2.25 (0.86) | 6.99 (0.81) | **0.0001** |
| PO_4_^-3^ | 0.23 (0.05) | 1.39 (0.08) | **0.0001** |
| DON | 21.79 (1.74) | 26.38 (7.87) | 0.2662 |
| N:P | 22.66 (6.14) | 7.51 (0.48) | **0.0051** |

^a^Tests are for t=1 d due to missing data for t=0 in June.
