# Supplementary material for: Dispersed Oil Disrupts Microbial Pathways in Pelagic Food Webs
Source: PLoS One. 2012 Jul 31;7(7):e42548. doi: 10.1371/journal.pone.0042548 (PMC3409195; doi:10.1371/journal.pone.0042548)
Supplement: Table S2 — P-values from MANOVAs carried out comparing the biomass of each group using Treatment as the factor and repeated measures analysis with time. (DOCX) [file pone.0042548.s007.docx]

**Table S2.** P-values from MANOVAs carried out comparing the biomass of each group using Treatment as the factor and repeated measures analysis with time.

|  | June | | August | |
| --- | --- | --- | --- | --- |
|  | Treatment | Time x Treatments | Treatment | Time x Treatments |
| Prokaryote Cells | **<0.0001** | **<0.0001** | **<0.0001** | **<0.0001** |
| Viruses | **<0.0001** | **0.0064** | **<0.0001** | **0.0021** |
| Dinoflagellates | **0.0006** | **<0.0001** | 0.6791 |  |
| Diatoms | **<0.0001** | **0.0007** | 0.0544 | 0.2485 |
| Ciliates | **<0.0001** | **0.0019** | **<0.0001** | **0.0003** |
| HNFs | 0.1732 |  | **0.0054** | **0.0014** |
