# Supplementary material for: Dispersed Oil Disrupts Microbial Pathways in Pelagic Food Webs
Source: PLoS One. 2012 Jul 31;7(7):e42548. doi: 10.1371/journal.pone.0042548 (PMC3409195; doi:10.1371/journal.pone.0042548)
Supplement: Table S3 — P-values from MANOVAs carried out as above for nutrients. (DOCX) [file pone.0042548.s008.docx]

**Supplemental Table 3.** P-values from MANOVAs carried out as above for nutrients.

|  | June | | August | |
| --- | --- | --- | --- | --- |
|  | Treatment | Time x Treatments | Treatment | Time x Treatments |
| NO_2_^-^ | **<0.0001** | **<0.0001** | **<0.0001** | **0.0002** |
| NO_3_^-^ | **0.0070** | **0.0071** | **<0.0001** | **0.0001** |
| NH_4_^+^ | **<0.0001** | **0.0141** | **<0.0001** | **<0.0001** |
| PO_4_^-3^ | **<0.0001** | **0.0008** | **<0.0001** | **<0.0001** |
| N:P | **<0.0001** | **0.0005** | **<0.0001** | **<0.0001** |
